# Regenerative Skin Remodeling by a Dual Hyaluronic Acid Hybrid Complex in Multimodal Preclinical Models

**DOI:** 10.3390/ijms27021027

**Published:** 2026-01-20

**Authors:** Hyojin Roh, Ngoc Ha Nguyen, Jinyoung Jung, Jewan Kaiser Hwang, Young In Lee, Inhee Jung, Ju Hee Lee

**Affiliations:** 1 Department of Dermatology & Cutaneous Biology Research Institute, Yonsei University College of Medicine, Seoul 03722, Republic of Koreanguyenngocha7996@gmail.com (N.H.N.);; 2 Mymirae Dermatologic Clinic, Seoul 07326, Republic of Korea; 3 Department of Dermatology, University of Medicine and Pharmacy at Ho Chi Minh City, Ho Chi Minh City 17000, Vietnam; 4 Mymirae Research Institute for Dermatologic Science, Seoul 07326, Republic of Korea; 5 Scar Laser and Plastic Surgery Center, Yonsei Cancer Hospital, Seoul 03722, Republic of Korea; 6 Global Medical Research Center Co., Ltd., Seoul 06526, Republic of Korea

**Keywords:** hyaluronic acid, rejuvenation, skin aging, antioxidants, melanogenesis

## Abstract

Skin aging arises from extracellular matrix degradation, inflammation, and pigmentation dysregulation, yet most existing rejuvenation strategies target only a subset of these processes. This study investigated the multimodal rejuvenation potential of a dual hyaluronic acid compound (DHC), composed of low- and high-molecular-weight HA integrated within a minimally cross-linked hybrid complex. In vitro assays using dermal fibroblasts, melanoma cells, and macrophages demonstrated that DHC enhanced fibroblast viability, collagen I/III and elastin production, antioxidant enzyme activity, and wound-healing capacity while reducing senescence markers. DHC markedly suppressed melanogenesis by downregulating the gene expression of *MITF*, *TYR*, and *TRP1*, and exerted strong anti-inflammatory activity by decreasing nitric oxide (NO) production and key cytokines, including TNF-α, IL-1β, IL-6, and CCL1. In a UVB-induced photoaging rat model, DHC reduced wrinkle depth, epidermal thickening, and melanin accumulation while improving elasticity, collagen density, hydration, and barrier integrity. Across these outcomes, DHC demonstrated biological effects that were comparable to, and in selected endpoints greater than, those of commonly used biostimulators and HA fillers in preclinical models. Collectively, these laboratory findings suggest that DHC exhibits broad preclinical bioactivity through combined biostimulatory, antioxidant, anti-inflammatory, and pigmentation-modulating effects. Further mechanistic and clinical studies are required to determine its translational relevance.

## 1. Introduction

Skin aging is characterized by the progressive degradation of the extracellular matrix (ECM), leading to the loss of collagen, elastin, and hyaluronic acid (HA). Clinically, this manifests as volume depletion, reduced elasticity, and wrinkle formation [1,2]. As demand for minimally invasive aesthetic procedures grows, modern rejuvenation strategies focus not only on volume restoration but also on reestablishing dermal architecture and stimulating endogenous tissue regeneration.

In this context, biostimulators, skin boosters, and dermal fillers have emerged as major modalities for facial rejuvenation. Among these, biostimulators such as poly-L-lactic acid (PLLA), poly-D, L-lactic acid (PDLLA), and calcium hydroxyapatite (CaHA) are designed to induce long-term dermal remodeling through fibroblast activation and subsequent neocollagenesis [3]. However, polymer-based biostimulators exert their effects primarily through controlled inflammatory pathways, which may be associated with delayed-onset adverse events, including nodule formation, fibrosis, or granulomatous reactions [4].

In contrast, skin boosters aim to improve skin elasticity, texture, and hydration by achieving uniform dermal distribution, thereby providing a more natural and gradual rejuvenation effect across a wide age range [5]. HA-based skin boosters, in particular, offer mild biostimulatory activity but are subject to relatively rapid enzymatic degradation by hyaluronidase [6]. Despite this limitation, HA has been widely adopted in aesthetic and dermatologic practice owing to its favorable biocompatibility, hygroscopic properties, and ability to restore volume and enhance skin hydration [7].

Beyond its role as a passive water-retaining matrix, HA is increasingly recognized as a biologically active molecule capable of modulating cellular behavior through receptor-mediated signaling pathways, such as CD44, as well as through anti-inflammatory and antioxidant mechanisms. Importantly, these biological effects are highly dependent on the molecular weight distribution and structural configuration of HA [8,9]. Conventional intradermal HA primarily improves skin quality, while its effects on pigmentation are variable and not routinely observed [10,11]. Notably, reports describing depigmenting effects in HA-related studies have often involved formulations incorporating additional bioactive components rather than HA alone [12,13], underscoring the need to interpret anti-melanogenic findings with caution within the broader context of the existing literature.

To address these challenges, the dual HA compound (DHC) was developed as a hyaluronic acid compound integrating three synergistic components: non-cross-linked low-molecular-weight HA (LMW-HA), non-cross-linked high-molecular-weight HA (HMW-HA), and a minimally cross-linked hybrid complex, stabilized with a trace amount of 1,4-butanediol diglycidyl ether (BDDE). This structure enables rapid hydration and antioxidant activity from LMW-HA [8], mechanical support and anti-inflammatory properties from HMW-HA [9], and prolonged volumizing and biostimulatory effects from the hybrid matrix [14,15]. Furthermore, in wound healing, combining LMW-HA, which drives early inflammatory response and cell migration, with HMW-HA, which later resolves inflammation and stabilizes the matrix, can enhance wound closure [16]. Theoretically, this synergy delivers both skin-booster and biostimulator benefits to maintain high biocompatibility and minimize inflammatory response, distinguishing it from traditional HA fillers, which contain only one or two of these components.

To validate this hypothesis, this study comprehensively evaluated DHC’s rejuvenating efficacy in comparison to other well-established biostimulators and fillers, using integrated in vitro and in vivo analyses. In vitro, its effects on cellular activity, ECM synthesis, inflammation, and melanogenesis were assessed using fibroblast, macrophage, and melanoma cell models. In vivo, a UVB-induced photoaging rat model was used to evaluate its impact on wrinkle formation, collagen deposition, skin barrier function, and hydration. Using these integrated preclinical models, the biological activity of DHC was contextualized against established HA-based products and biostimulators to clarify its position within the broader landscape of HA research in skin rejuvenation and pigmentation regulation.

## 2. Results

### 2.1. In Vitro Study Results

#### 2.1.1. Cell Viability

DHC demonstrated high cell viability in Human dermal fibroblasts (HDFs) across all tested concentrations (0.125–1%), maintaining survival rates higher than that of the untreated control (*p* < 0.05; Figure S1A). The survival rate of melanoma cells also remained stable across all concentrations, significantly higher than in the control group (*p* < 0.05; Figure S1B). In macrophages, DHC exhibited mild proliferation-promoting effects at higher concentrations (1%), comparable to the control group (Figure S1C). From these outcomes, we chose the highest concentration without cytotoxicity, which was 1%, for all test products for subsequent experiments.

Over time, DHC at 1% concentration significantly increased HDFs proliferation in a time-dependent manner, reaching a peak at 72 h, surpassing that of the control group (*p* < 0.05; Figure S1D).

#### 2.1.2. ECM Protein Synthesis

**Figure 1:**
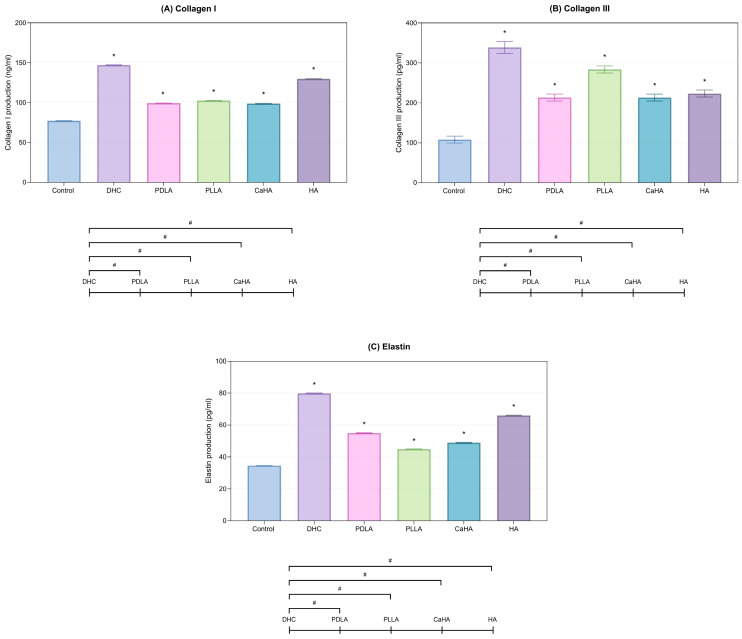
Collagen and elastin production in HDFs treated with DHC and other comparators. (**A**) Collagen I, (**B**) Collagen III, and (**C**) Elastin levels were quantified by ELISA. Values are expressed as mean ± SD. * *p* < 0.05 vs. control; # *p* < 0.05 between groups.

DHC significantly enhanced collagen I and collagen III synthesis compared to the control group (*p* < 0.05; Figure 1A,B). Moreover, elastin production was markedly upregulated compared to the control (*p* < 0.05; Figure 1C), indicating improved dermal elasticity.

Comparative analysis revealed that DHC induced the highest overall ECM protein expression among all tested products (*p* < 0.05, Figure 1A–C).

#### 2.1.3. Antioxidant, Anti-Aging, and Wound Healing Efficacy

**Figure 2:**
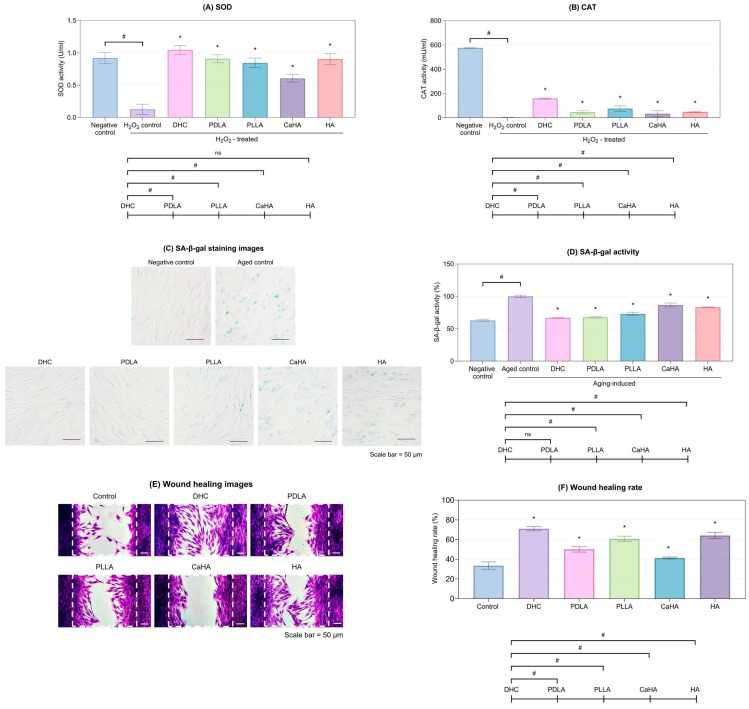
Antioxidant enzyme activities of (**A**) SOD and (**B**) CAT activities in H_2_O_2_-treated HDFs were measured after treatment with DHC and other comparators. SA-β-galactosidase staining images (**C**) and quantitative activity (**D**) in aging-induced HDFs treated with DHC and other comparators. Wound healing rate images (**E**) and quantitative analysis (**F**) in HDFs treated with DHC and other comparators. The white dashed box indicates the wound gap created by the culture insert. Values are expressed as mean ± SD. * *p* < 0.05 vs. control; # *p* < 0.05 between groups; ns, not significant.

DHC significantly restored both superoxide dismutase (SOD) and catalase (CAT) activities in HDFs compared with the oxidative-stress controls (*p* < 0.05; Figure 2A,B). Among all tested products, DHC demonstrated the strongest antioxidative performance (*p* < 0.05).

Aging-induced HDFs (Aged control) exhibited high Senescence-Associated beta-Galactosidase (SA-β-gal) expression, whereas DHC treatment markedly reduced SA-β-gal activity to levels comparable to the negative control (*p* < 0.05; Figure 2C,D). The degree of reduction exceeded that observed for most commercial products.

In addition, DHC treatment achieved a significantly higher wound-healing rate in HDFs compared with both the control and all other comparators (*p* < 0.05; Figure 2E,F).

#### 2.1.4. Inhibition of Melanin Production and Melanogenesis Gene Expression

**Figure 3:**
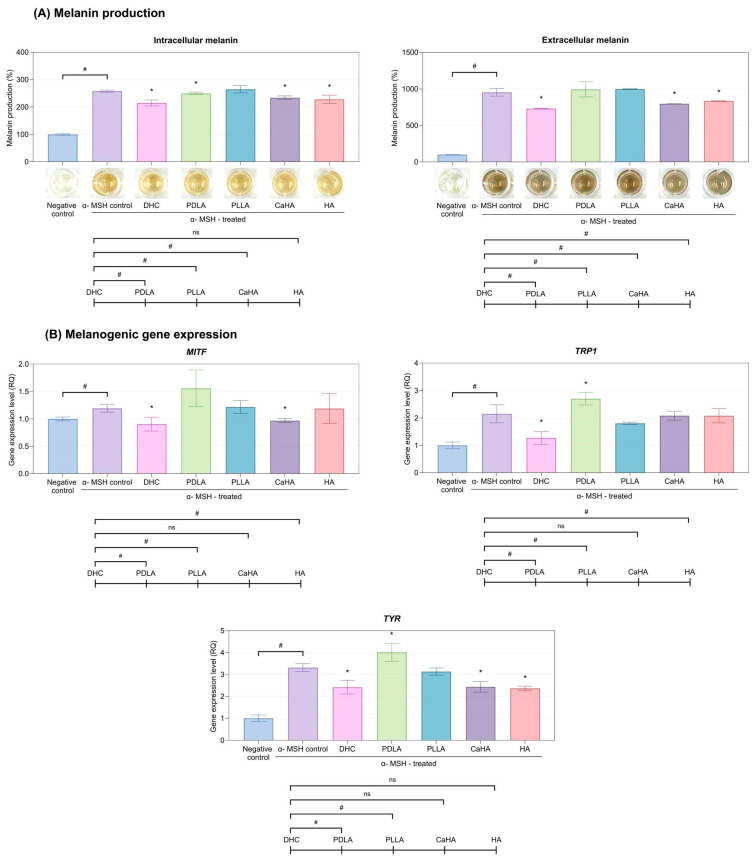
(**A**) Intracellular and extracellular melanin levels of mouse melanoma cells after treatment with DHC and other comparators. (**B**) Expression of melanogenic genes—*MITF*, *TYR*, and *TRP1*—of mouse melanoma cells after treatment with DHC and other comparators. Values are expressed as mean ± SD. * *p* < 0.05 vs. control; # *p* < 0.05 between groups; ns, not significant.

Alpha-melanocyte-stimulating hormone (α-MSH) stimulation markedly increased both intra- and extracellular melanin levels in mouse melanoma cells compared with the negative control. DHC treatment significantly reduced melanin content in both compartments relative to the α-MSH–treated control. (*p* < 0.05, Figure 3A). Among all products tested, DHC displayed the most pronounced melanin-reducing activity (*p* < 0.05).

In mouse melanoma cells, DHC treatment significantly downregulated the melanogenesis-related genes: *Microphthalmia-associated Transcription Factor (MITF)*, *tyrosinase (TYR)*, and *Tyrosinase-related protein 1 (TRP1)* compared with the α-MSH control (*p* < 0.05; Figure 3B). Relative to other comparators, DHC exhibited a more consistent inhibitory profile across all three genes, suggesting that its melanin-reducing effect is mediated primarily through modulation of the MITF–TYR signaling axis (*p* < 0.05).

#### 2.1.5. Evaluation of Inflammatory Response

**Figure 4:**
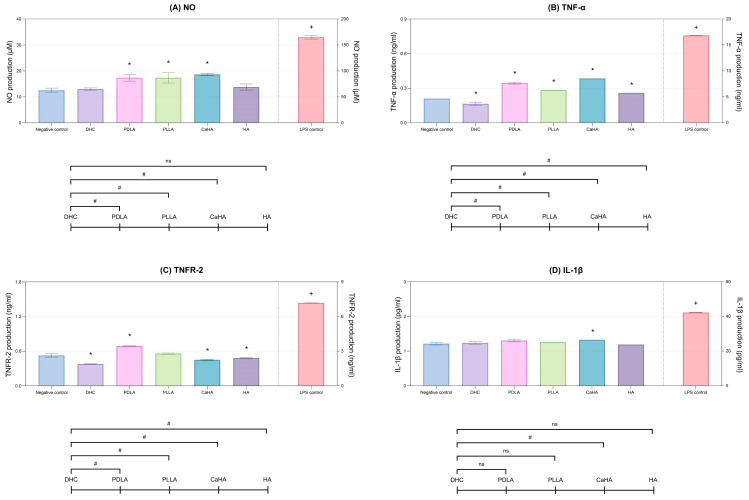
Inflammatory response of DHC in mouse macrophages. (**A**) Nitric oxide (NO), (**B**) TNF-α, (**C**) TNFR-2, and (**D**) IL-1β production were quantified after treatment with DHC, other comparators, and LPS (positive control). Values are expressed as mean ± SD. * *p* < 0.05 vs. LPS control; + *p* < 0.05 vs. negative control; # *p* < 0.05 between groups; ns, not significant.

Lipopolysaccharide (LPS) induces the strongest inflammatory response, while DHC significantly suppressed the production of Tumor Necrosis Factor-alpha (TNF-α) and Tumor Necrosis Factor Receptor 2 (TNFR-2) compared with the control (*p* < 0.05; Figure 4A,B), and maintaining nitric oxide (NO) and Interleukin (IL)-1β levels comparable to the control (*p* > 0.05; Figure 4C,D). Although all comparators exhibited mild anti-inflammatory activity, DHC consistently maintained the lowest levels of NO and TNF-α (*p* < 0.05; Figure 4A–C).

#### 2.1.6. Mitigation of LPS-Induced Inflammation

**Figure 5:**
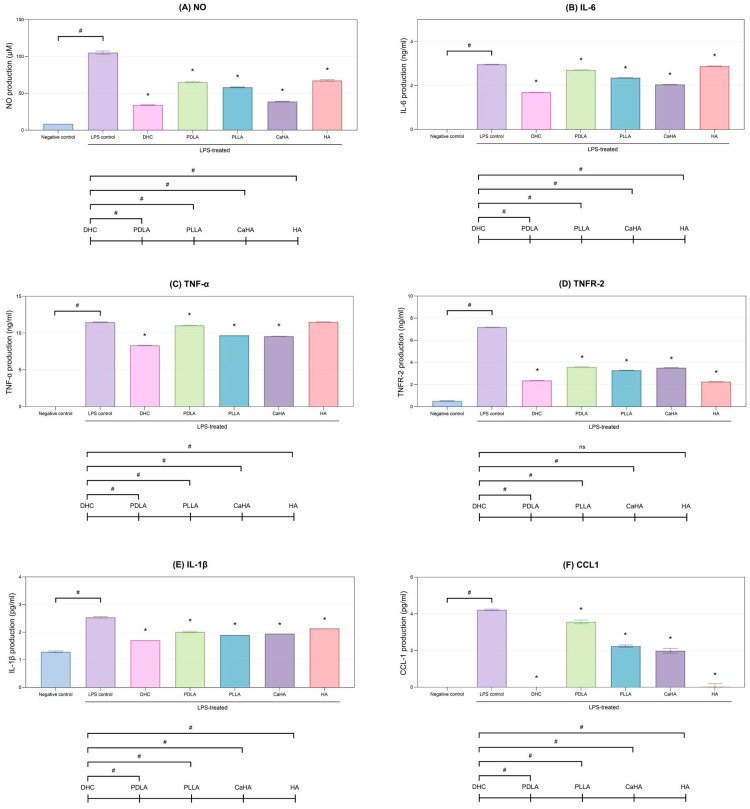
Mitigating effects of DHC on LPS-induced inflammation in mouse macrophages. Following LPS stimulation, the levels of (**A**) NO, (**B**) IL-6, (**C**) TNF-α, (**D**) TNFR-2, (**E**) IL-1β, and (**F**) CCL1 were assessed after treatment with DHC and other comparators. Values are expressed as mean ± SD. * *p* < 0.05 vs. LPS control; # *p* < 0.05 between groups, ns, not significant.

DHC treatment markedly reduced all pro-inflammatory markers (NO, IL-6, TNF-α, IL-1β, TNFR-2, Chemokine (C-C motif) ligand 1 (CCL1)) compared with the LPS control in mouse macrophages (*p* < 0.05; Figure 5A–F). Furthermore, it showed the strongest suppression among all tested products (*p* < 0.05; Figure 5A–F).

### 2.2. In Vivo Study on a UVB-Irradiated Rat Model

After comparative evaluation of multiple candidate comparators alongside DHC in the in vitro studies, PLLA and HA—representing two of the most widely established classes of dermal volumizing materials—were selected as reference comparators for the subsequent in vivo experiments.

#### 2.2.1. Antioxidant Activity

**Figure 6:**
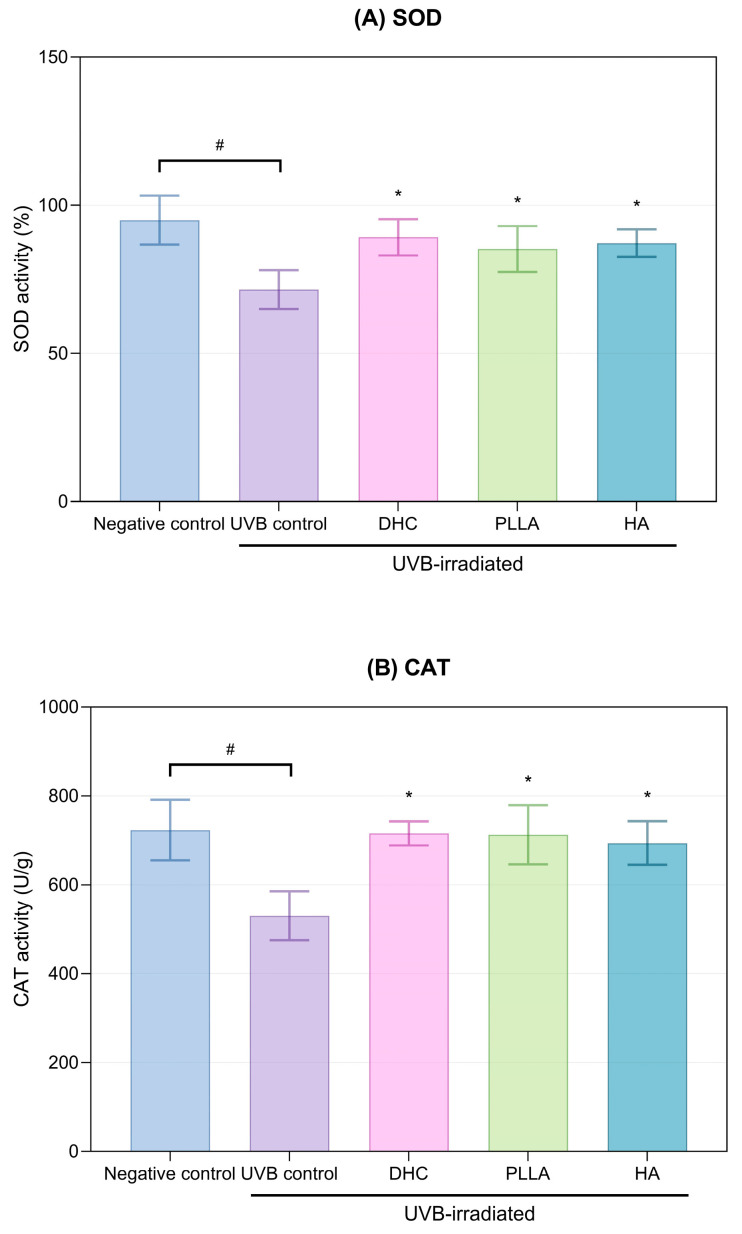
Antioxidant enzyme activity of DHC in UVB-irradiated rat skin. (**A**) SOD and (**B**) CAT activities were measured after UVB exposure and subsequent treatment with DHC, PLLA, and HA. Values are expressed as mean ± SD. * *p* < 0.05 vs. UVB control; # *p* < 0.05 vs. negative control.

Ultraviolet B (UVB) exposure significantly reduced SOD and CAT activities in skin tissue (*p* < 0.05; Figure 6A,B). Treatment with DHC effectively restored both antioxidant enzymes compared to UVB control (*p* < 0.05; Figure 6A,B). The recovery was also comparable to PLLA and HA (*p* > 0.05).

#### 2.2.2. Anti-Inflammatory Response

**Figure 7:**
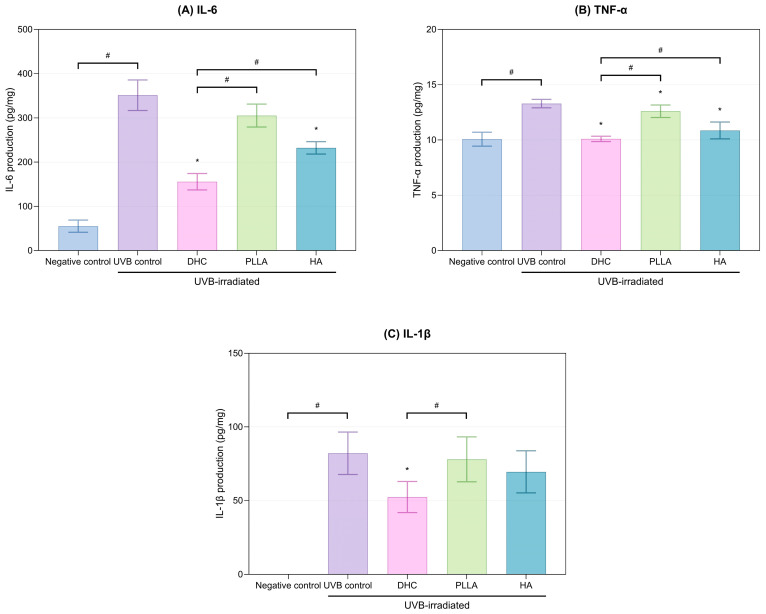
Suppression of UVB-induced pro-inflammatory cytokines in rat skin. (**A**) IL-6, (**B**) TNF-α, and (**C**) IL-1β levels were measured after UVB irradiation and subsequent treatment with DHC, PLLA, and HA. Values are expressed as mean ± SD. * *p* < 0.05 vs. UVB control; # *p* < 0.05 between groups.

UVB irradiation markedly elevated pro-inflammatory cytokines (IL-6, TNF-α, and IL-1β) compared with the negative control (*p* < 0.05, Figure 7A–C). Conversely, DHC significantly reduced all three cytokines, achieving greater suppression than PLLA and HA (*p* < 0.05; Figure 7A–C).

#### 2.2.3. Histological Evaluation

**Figure 8:**
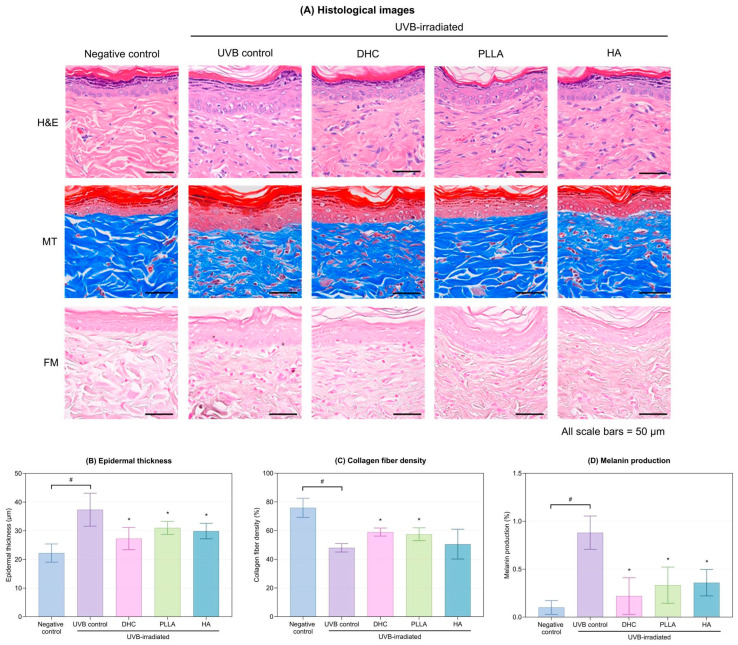
Histological analysis in the UVB-irradiated rat model. (**A**) Representative Hematoxylin and Eosin (H&E), Masson’s trichrome (MT), and Fontana-Masson (FM) staining images. (**B**) epidermal thickness, (**C**) collagen fiber density, and (**D**) melanin production were quantified after UVB exposure and subsequent treatment with DHC, PLLA, and HA. Values are expressed as mean ± SD. * *p* < 0.05 vs. UVB control; # *p* < 0.05 between groups.

Histological staining revealed that UVB exposure caused collagen loss, epidermal thickening, and increased melanin deposition. DHC markedly reversed these alterations, showing normalized epidermal thickness in Hematoxylin and Eosin (HE) staining, denser collagen bundles in Masson’s Trichrome (MT) staining, and reduced melanin in the basal layer in Fontana-Masson (FM) staining (Figure 8A). Quantitative assessment further confirmed these findings: collagen fiber density increased significantly, whereas melanin content and epidermal thickness were markedly reduced compared with the UVB control (*p* < 0.05; Figure 8B–D). These effects were comparable to those of PLLA and HA.

#### 2.2.4. Wrinkle Improvement, Elasticity Recovery, and Collagen I Production

**Figure 9:**
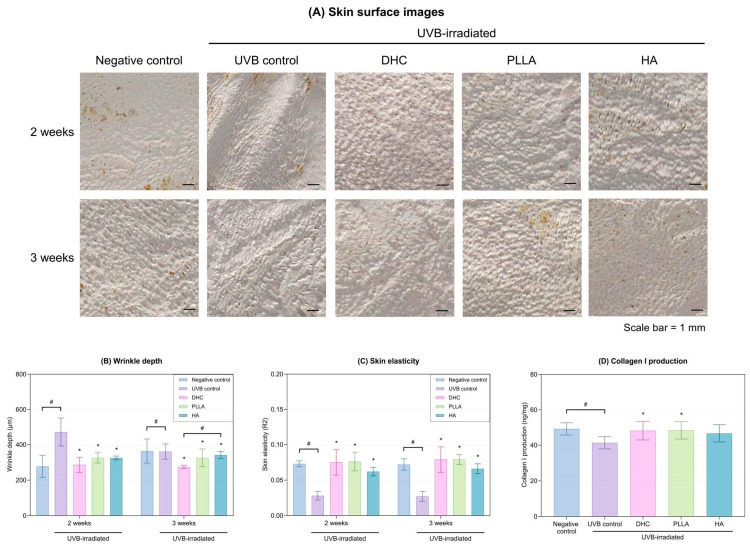
Macroscopic and quantitative evaluation of the anti-photoaging efficacy of DHC. (**A**) Representative skin surface images at 2 and 3 weeks following injection with DHC, PLLA, and HA. (**B**) Wrinkle depth and (**C**) skin elasticity were measured at 2 and 3 weeks post-UVB exposure and subsequent treatment with DHC, PLLA, and HA. (**D**) Collagen I protein levels were quantified using ELISA. Values are expressed as mean ± SD. * *p* < 0.05 vs. UVB control; # *p* < 0.05 between groups.

Surface imaging showed that DHC treatment visibly reduced wrinkle formation compared to the UVB control after 2 weeks and maintained its effectiveness for up to 3 weeks of exposure (Figure 9A). Correspondingly, wrinkle depth decreased, and skin elasticity significantly improved (*p* < 0.05 vs. UVB control; Figure 9B,C). Collagen I content also increased relative to UVB control (*p* < 0.05 vs. UVB control; Figure 9D). Furthermore, DHC demonstrated outcomes comparable to those of the other comparators.

#### 2.2.5. Skin Barrier Function and Hydration

**Figure 10:**
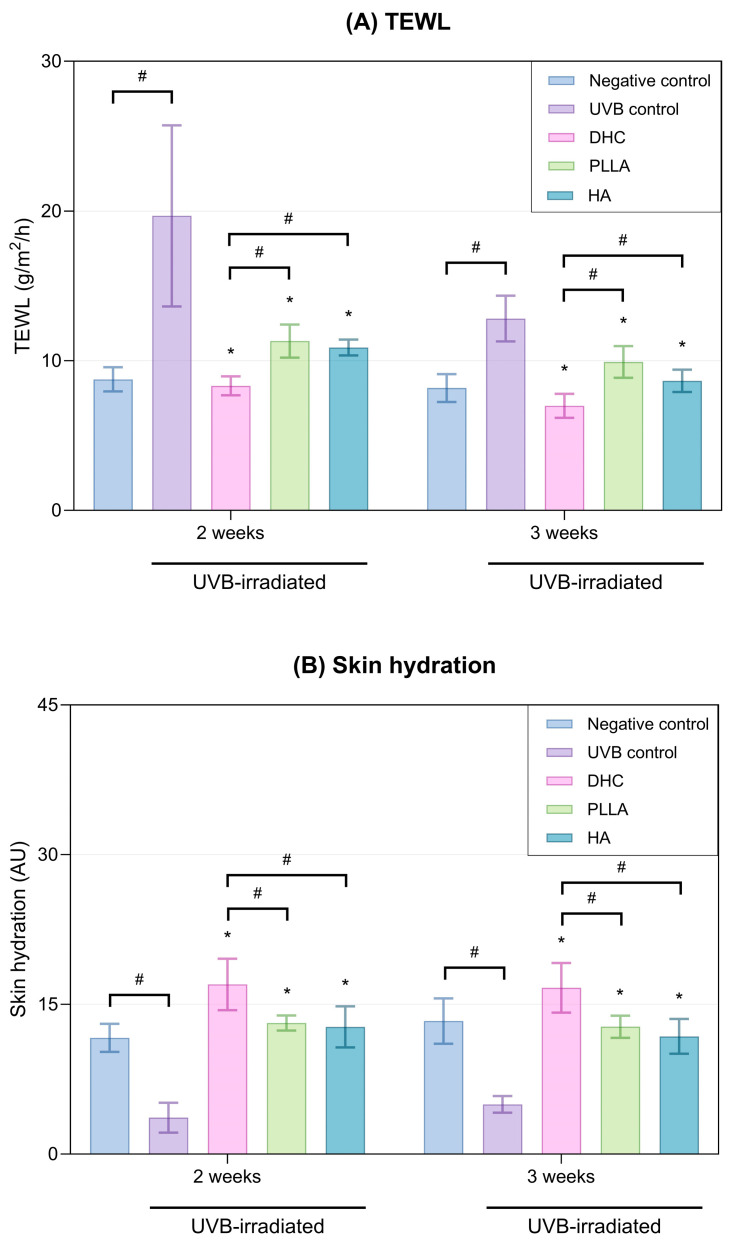
Effect of DHC on skin barrier function in UVB-irradiated rat model. (**A**) Transepidermal water loss (TEWL) and (**B**) Skin hydration were measured at 2 and 3 weeks after UVB exposure and subsequent treatment with DHC, PLLA, and HA. Values are expressed as mean ± SD. * *p* < 0.05 vs. UVB control; # *p* < 0.05 between groups.

UVB irradiation significantly increased transepidermal water loss (TEWL) and reduced skin hydration. DHC treatment effectively lowered TEWL and enhanced hydration throughout 3 weeks (*p* < 0.05 vs. UVB control; Figure 10A,B). These improvements were better in comparison to both PLLA and HA at all time points (*p* < 0.05).

## 3. Discussion

This study demonstrated that DHC, a novel hyaluronic acid compound, provides comprehensive rejuvenation through multiple synergistic biological mechanisms. The effects of DHC were validated through integrated in vitro and in vivo analyses, including antioxidant, anti-inflammatory, hydration-enhancing, and pigmentation-modulating activities. This multi-modal efficacy may be associated with the combination of LMW-HA and HMW-HA within a minimally cross-linked hybrid complex, which could represent a potential advantage compared with existing formulations in aesthetic and regenerative medicine. However, several limitations exist in our study, including protein-level confirmation of key signaling nodes (e.g., MITF, TYR, or CD44-associated downstream pathways) or using formulation-matched controls (e.g., mixture of LMW-HA and HMW-HA without hybrid cross-linking) as a comparator. These considerations underscore that the present findings should be interpreted as demonstrating biological plausibility and comparative preclinical performance, rather than definitive mechanistic superiority.

HMW-HA is well-known for its anti-inflammatory and tissue-protective benefits, primarily through CD44-mediated signaling, which maintains hydration and structural integrity of the skin [9,17,18]. LMW-HA, on the other hand, is recognized for its antioxidant properties and its ability to enhance fibroblast activity, improving skin hydration and elasticity [8,19]. When combined in DHC, these two forms of HA not only provide immediate hydration but also induce long-term skin remodeling by enhancing collagen synthesis, improving skin elasticity, and reducing other signs of aging. The addition of the minimally cross-linked hybrid complex further stabilizes the formulation, enhancing its sustained effects and providing structural reinforcement to the skin [14,15,20]. Unlike conventional HA fillers, which often rely on single-molecule components or highly cross-linked structures, DHC’s multi-component structure provides a balance between rapid, superficial hydration and deeper, longer-lasting dermal remodeling, making it an innovation in the field of skin rejuvenation.

With aging and oxidative stress, dermal fibroblasts experience reduced proliferation, increased senescence, and diminished ECM synthesis, resulting in dermal thinning, laxity, and wrinkle formation [21]. To counteract these changes, animal skin studies applying LMW-HA, HMW-HA, or cross-linked HA have observed an enhanced collagen I and III production and promoted wound repair [20,22]. In our study, DHC enhanced collagen production and wound healing to reverse the aforementioned aging symptoms while maintaining low inflammatory cytokine levels, suggesting that its biostimulatory effects are not primarily mediated by classical foreign body–type inflammation. One plausible explanation is mechanotransductive signaling, in which the mechanical strain of the dermis due to the HA injection stretches dermal fibroblasts and subsequently activates them to produce ECM components [20], while LMW-HA provides a direct antioxidant effect on dermal fibroblasts for rejuvenation [8,19]. Furthermore, HA can directly influence fibroblast migration and proliferation via interaction with CD44 and Receptor for hyaluronan-mediated motility, as well as modulate MMP activity to prevent excessive ECM degradation [23]. Finally, incorporating both LMW-HA, which can induce mild inflammation and cell migration in the early phase of tissue repair, and HMW-HA, which alleviates inflammation and promotes ECM remodelling, can support wound healing for fibroblasts [16]. The hybrid architecture of DHC may therefore enable collagen induction through a combination of mechanical stimulation and receptor-mediated signaling, decoupled from sustained inflammatory activation. While these mechanisms are biologically plausible and supported by prior studies, direct interrogation of mechanotransductive and CD44-dependent pathways was beyond the scope of the present work and warrants further investigation.

Melanogenesis, regulated by *MITF*, governs the expression of *TYR* and *TRP1*, enzymes central to melanin biosynthesis [24]. Excessive activation increases intracellular and extracellular melanin accumulation, contributing to hyperpigmentation and uneven skin tone. In the context of pigmentation regulation, DHC consistently reduced melanin production and downregulated *MITF*-associated melanogenic gene expression in both α-MSH–stimulated melanoma cells and UVB-irradiated rat skin. Previous studies examining HA-based injectables have generally reported neutral or indirect effects on pigmentation [10,11], or have relied on combination formulations incorporating additional depigmenting or antioxidant agents [12,13]. Within this existing body of literature, the present findings suggest that a multi-component HA-only formulation, when designed to integrate distinct molecular weights and sustained tissue residence, may exert measurable modulatory effects on melanogenesis-related signaling pathways. These effects are likely mediated indirectly through attenuation of oxidative stress and inflammatory cues known to activate MITF signaling, rather than through direct melanocyte-targeted inhibition [8,17,25,26]. However, further mechanistic validation will be required to delineate these pathways more precisely.

Conventional biostimulators such as PLLA, PDLLA, and CaHA rely on controlled inflammation to stimulate collagen production [3], but this mechanism also carries risks such as nodules, fibrosis, and prolonged swelling [4]. In contrast, DHC promotes neocollagenesis while maintaining low baseline cytokine levels and suppressing their release under LPS-induced inflammation. This supports DHC’s biocompatibility, low immunogenicity, potentiating it as a non-inflammatory biostimulator with minimal adverse tissue reactions in laboratory conditions. Future clinical studies are needed to translate these outcomes into human participants.

Within the skin, HA plays an integral part in regulating barrier function and moisture retention, mainly due to its ability to hold water 1000 times its own weight [27]. With over 50% of the body’s HA content is contained in the integumentary system, about 25 µg/g of HA resides in the epidermis, while 120–200 µg/g is in the dermis. Both types of HA are humectants, but epidermal HA also modulates keratinocyte activity and maintains skin barrier integrity via CD44 interaction, preventing excessive TEWL [28]. Meanwhile, aging and chronic UV exposure can reduce the HA content and compromise the skin barrier, thereby changing the TEWL, decreasing hydration, and thickening the stratum corneum [2,29,30]. In our photoaging rat model, DHC significantly reversed these detrimental effects. This is consistent with prior clinical studies of combined LMW-HA and HMW-HA formulations, which improved skin hydration and reduced TEWL through direct supplementation of HA molecules [31,32]. Together, these findings explain the strong ability of DHC to enhance effective barrier repair and skin homeostasis in experimental preclinical models, and additional clinical studies are warranted to confirm these findings.

While this study provides robust preclinical evidence for the multi-dimensional rejuvenating effects of DHC, several limitations should be acknowledged. Although multiple biological endpoints were evaluated, the mechanistic interpretations remain largely associative, as anti-melanogenic and biostimulatory effects were inferred primarily from antioxidant, anti-inflammatory, and transcriptional changes without direct pathway-level or protein-based validation, namely immunohistochemistry or Western blot. Similarly, the classification of DHC as a non-inflammatory biostimulator is based on cytokine suppression rather than detailed intracellular signaling analyses. In addition, although DHC showed favorable performance compared with several established biostimulators and HA products, the absence of a direct head-to-head comparison with non-animal stabilized hyaluronic acid (NASHA)-type fillers or a simple, non-cross-linked physical blend of LMW-HA and HMW-HA limits definitive attribution of its advantages to its hybrid architecture. Furthermore, while the UVB-induced dorsal rat skin model is appropriate for mechanistic exploration of photoaging, it does not fully replicate human facial skin aging or clinical injection practices; therefore, the findings should be interpreted as demonstrating preclinical biological potential rather than clinical efficacy. Finally, given the small sample size and the exploratory and hypothesis-generating nature of the study, adjustments for multiple comparisons were not applied to control type I errors (false positives).

From a translational perspective, the present findings support a stepwise framework for future investigation. First, mechanistic validation studies should be conducted to confirm the involvement of specific signaling pathways implicated in the observed effects, including CD44-mediated fibroblast activation, mechanotransductive responses, and MITF-associated melanogenic regulation, using protein-level analyses and pathway inhibition models. Second, formulation-controlled comparative studies employing matched HA blends will be necessary to isolate the contribution of hybrid HA architecture. Human ex vivo skin models may provide an intermediate platform to evaluate dermal distribution, pigmentation responses, and inflammatory markers under clinically relevant injection conditions. Finally, early-phase clinical studies should focus on safety, tolerability, and biological readouts—such as skin hydration, barrier function, pigmentation indices, and biomarker changes—rather than direct efficacy claims. These sequential steps will be essential to determine whether the broad preclinical bioactivity observed for DHC translates into durable and clinically meaningful outcomes.

## 4. Materials and Methods

### 4.1. Test Product

The studies employed DHC 1% (Hilowave, Higher Corporation Co., Ltd., Daegu, Republic of Korea) to evaluate the efficacy and functional properties of the test product. Other comparators included PDLA (Juvelook, Vaim Global Inc., Seoul, Republic of Korea), PLLA (Sculptra, Galderma, Zug, Switzerland), CaHA (Radiesse, Merz Aesthetics, Raleigh, NC, USA), and HA (Skinvive, Allergan Aesthetics, Irvine, CA, USA).

### 4.2. In Vitro Study

#### 4.2.1. Cell Culture

HDFs (Thermo Fisher Scientific, Waltham, MA, USA), mouse melanoma cells (B16F10, ATCC, Manassas, VA, USA), and mouse macrophages (RAW 264.7, ATCC) were cultured in DMEM (Lonza, Basel, Switzerland) supplemented with 10% FBS (Gibco, Waltham, MA, USA) and 1% penicillin-streptomycin (Gibco, Waltham, MA, USA) at 37 °C in a humidified 5% CO_2_ incubator.

#### 4.2.2. Cell Viability Evaluation

HDFs, mouse melanoma cells, and mouse macrophages were seeded at 1 × 10^4^ cells/well in a 96-well plate and cultured until reaching 80% confluence. DHC and other comparators were then applied with different concentrations (0.125%, 0.25%, 0.5%, 1%), and the cells were incubated for 24 hours (h). To evaluate the effects on HDF proliferation, DHC and other comparators were added to HDF cultures at a concentration of 1% for 24, 48, and 72 h. Subsequently, WST substrate solution (CCK-8, Dojindo, Rockville, MD, USA) was added to each well, followed by incubation at 37 °C for 2 h. The optical density (OD) was measured at 450 nm using a microplate reader (VARIOSKAN LUX, Thermo Fisher Scientific). Cell viability was calculated from OD values, with higher absorbance indicating greater cell viability.

#### 4.2.3. ECM Protein Evaluation

HDFs were seeded at 5 × 10^4^ cells/well in 6-well plates, cultured at 37 °C with 5% CO_2_ to over 80% confluence, and treated with DHC and other comparators for 24 h. The culture medium was centrifuged at 2000× *g* for 10 min, and the supernatant was used for analysis, excluding cell debris. Protein concentration was measured using the Bicinchoninic Acid (BCA) Protein Assay Kit (Sigma-Aldrich, St. Louis, MO, USA). Enzyme-Linked Immunosorbent Assay (ELISA) was designed to quantify the production of wrinkle-improving proteins using: Human Pro-Collagen I α1 SimpleStep ELISA Kit (ab210966, Abcam, Cambridge, MA, USA), Human Collagen Type III Alpha 1 ELISA Kit (NBP2-75858, Novus Biologicals, Centennial, CO, USA), Human Elastin ELISA Kit (MBS704171, MyBioSource, San Diego, CA, USA).

All procedures followed the manufacturers’ protocols. OD was measured using the VARIOSKAN LUX reader and protein concentrations were derived from the standard curve regression equation. Higher OD values correspond to greater protein production.

#### 4.2.4. Evaluation of SA-β-Gal Activity

This assay was conducted to assess SA-β-gal activity, a marker of cellular senescence, following treatment with the test product.

HDFs were seeded at 5 × 10^4^ cells/well in 24-well plates and passaged more than 30 times to induce natural senescence. Upon reaching approximately 80% confluence, senescent cells were treated with DHC and other comparators for 24 h. After incubation, cells were washed with PBS, fixed with a fixation solution, and stained with X-gal staining solution at 37 °C for 24 h.

Stained cells were observed under an optical microscope (BX43F; OLYMPUS, Tokyo, Japan) at 200× magnification, and the percentage of X-gal–positive (blue-stained) cells relative to the total cell count was quantified. A higher percentage indicates increased cellular senescence.

#### 4.2.5. Assessment of Antioxidant Enzyme Activity

To assess antioxidant enzyme activity, HDFs were seeded at 5 × 10^4^ cells/well in 6-well plates, cultured to roughly 80% confluence, and treated with DHC and other comparators for 24 h. The supernatant was then collected after centrifugation at 2000× *g* for 10 min.

OxiTec™ SOD and CAT Assay Kits (BIOMAX, Seoul, Republic of Korea) were used per manufacturer’s instructions, and OD was measured using the VARIOSKAN LUX reader. Lower absorbance corresponds to higher enzymatic activity.

#### 4.2.6. Wound Healing Assay

To assess the regenerative capacity of the test product, HDFs were seeded at 5 × 10^4^ cells/well into an IBIDI Culture Insert (Ibidi GmbH, Bavaria, Germany) consisting of three reservoirs separated by 500 ± 100 μm barriers. Upon reaching approximately 90% confluence, the insert was carefully removed to create a uniform wound gap. DHC and other comparators at 1% concentration were then applied as designated.

Following incubation for 24 h, cells were gently washed with PBS, fixed in 4% paraformaldehyde, and stained with 0.2% crystal violet. Cell migration and wound closure were examined under a phase-contrast microscope (Primovert; Carl Zeiss, Oberkochen, Germany) at 100× magnification.

Quantitative analysis of wound closure was performed using ImageJ software version 1.53 (NIH, Bethesda, MD, USA) according to the following equation:

Wound recovery rate (%) = (0 h wound area − 48 h wound area) × 100/0 h wound area

#### 4.2.7. Evaluation of Melanin Production and Melanogenesis Gene Expression

Mouse melanoma cells were seeded at 1 × 10^5^ cells/well in a 6-well plate, cultured to approximately 80% confluence. The cells were simultaneously treated to α-MSH (100 nM) and DHC or other comparators for 72 h in phenol red–free DMEM (Gibco).

- For intracellular melanin, the culture medium was removed, and each well was treated with 250 µL of 1 M sodium hydroxide (DAEJUNG, Siheung, Republic of Korea) containing 10% DMSO (Sigma-Aldrich). The samples were incubated at 60 °C for 10 min in the dark, scraped, and subsequently heated at 95 °C for 30 min. After centrifugation at 4000× *g* for 10 min, the resulting supernatant was collected for analysis. For extracellular melanin, the culture medium was centrifuged at 4000× *g* for 10 min, and the supernatant was collected. OD was measured at 475 nm using the VARIOSKAN LUX reader and melanin content was calculated according to the analytical protocol. Total protein concentration was determined using the BCA Protein Assay Kit (Sigma-Aldrich) for normalization.
- Real-Time Polymerase Chain Reaction (RT-PCR): Total RNA was extracted using the RNeasy Mini Kit (Qiagen, Hilden, Germany), and complementary DNA (cDNA) was synthesized with the RNA to cDNA EcoDry™ Premix (Oligo dT) (Clontech, Mountain View, CA, USA). Real-time PCR was performed using TaqMan Fast Advanced Master Mix (Applied Biosystems, Foster City, CA, USA) with primers for microphthalmia-associated transcription factor (MITF, Mm00434954_m1, Applied Biosystems), tyrosinase-related protein 1 (TRP1, Mm00453201_m1, Applied Biosystems), tyrosinase (TYR, Mm00495817_m1, Applied Biosystem). GAPDH (Mm99999915_g1, Applied Biosystems) served as the internal control. Ct values were obtained from amplification curves, and relative quantification was calculated, where lower Ct values indicate higher gene expression.

#### 4.2.8. Inflammation Evaluation

NO assay: To assess a key inflammatory mediator released by activated macrophages, mouse macrophages were seeded at 1 × 10^6^ cells/well in 12-well plates, cultured to around 80% confluence, and treated with DHC, other comparators, and LPS (1 μg/mL) for 24 h. The culture medium was then centrifuged at 2000× *g* for 10 min, and the supernatant was mixed with Griess reagent (Sigma-Aldrich) at a 1:1 ratio and incubated at room temperature. OD was measured at 540 nm using the VARIOSKAN LUX reader. NO concentrations were calculated from a sodium nitrite (NaNO_2_) standard curve, where lower absorbance values indicated reduced NO production and greater anti-inflammatory efficacy of the test product.

ELISA: Mouse macrophages were seeded into 6-well plates at 1 × 10^6^ cells/well and cultured until reaching approximately 80% confluence.

- To assess inflammatory responses, DHC, other comparators, or LPS (positive control) were applied to the respective treatment groups for 24 h. Following incubation, the culture media were collected, centrifuged at 2000× *g* for 10 min, and the resulting supernatants were analyzed using: Mouse TNF-α SimpleStep ELISA Kit (ab208348, Abcam), sTNF RII (TNFRSF1B) Mouse SimpleStep ELISA Kit (ab202412, Abcam), Mouse IL-1β SimpleStep ELISA Kit (ab197742, Abcam).
- To assess the anti-inflammatory effects, the cells were first treated with LPS (1 μg/mL) and subsequently co-treated with DHC and other comparators for 24 h. After incubation, the culture medium was collected, centrifuged, and the supernatants were used for cytokine quantification via: Mouse IL-6 ELISA Kit (M600B-1, R&D Systems, Minneapolis, MN, USA), Mouse TNF-α SimpleStep ELISA Kit (ab208348, Abcam), sTNF RII (TNFRSF1B) Mouse SimpleStep ELISA Kit (ab202412, Abcam), Mouse IL-1β SimpleStep ELISA Kit (ab197742, Abcam), TCA-3 (CCL1) Mouse ELISA Kit (ab155460, Abcam).

### 4.3. In Vivo Study

#### 4.3.1. Animal Housing and Experimental Design

Male Sprague-Dawley rats (6 weeks old) were obtained from RaonBio Co., Ltd. (Yongin, Republic of Korea) and acclimated for 1 week prior to experimentation. Sprague-Dawley rats have relatively thick and wide skin, which makes it easier to observe histological changes after UV exposure. The dermal layer of these rats shows clear changes in collagen and elastin density, making them suitable for assessing photoaging markers. Additionally, the rats have a moderate distribution of melanin, which facilitates the evaluation of pigmentation changes, wrinkles, and elasticity alterations.

Animals were housed under controlled conditions (24 ± 0.5 °C, 55–65% humidity, 12 h light/dark cycle) with free access to rodent chow and water and monitored for adverse events. The animal studies were performed after receiving approval of the Institutional Animal Care and Use Committee (IACUC) in Hulux (IACUC approval No. Hulux-2025-05-001, 16 May 2025).

After acclimation, rats were weighed and randomly divided into five groups. Each group consisted of 6 rats, which was determined based on a similar study using a photoaging SD rat model to evaluate the efficacy of a novel filler product [33]. This minimized initial weight differences between groups and ensured a balanced distribution of variability among individuals. The treatment and sample measurement order was assigned randomly to minimize the potential influence of time-of-day effects. In addition, the animal cages were randomly assigned at the start of the experiment and maintained in the same location throughout the study to ensure uniform environmental conditions. To further reduce external variability, consistent rearing conditions, including temperature, lighting, water, and feed, were strictly maintained. These measures were implemented to minimize factors that could potentially influence the evaluation of the treatment effects.

UVB irradiation was delivered using four UVB lamps (G20T10E, SANKYO DENKI, Kanagawa, Japan; peak wavelength 312 nm), and the UVB irradiation system was configured by Hulux (Seongnam, Republic of Korea) with controlled parameters, including irradiation distance and intensity. Under isoflurane anesthesia, the dorsal skin—except in the negative control group—was exposed to UVB for 8 weeks. The exposure dose was gradually increased: 60 mJ/cm^2^/day (weeks 1–2), 120 mJ/cm^2^/day (week 3), 180 mJ/cm^2^/day (week 4), and 240 mJ/cm^2^/day (weeks 5–8), administered five times per week under consistent conditions. The UVB dose was calibrated using a UV radiometer prior to irradiation to ensure consistency and reproducibility. The distance between the UVB lamps and the skin surface of the animals was fixed at approximately 20 cm within the cage. UV intensity was measured using a UV meter (UV-340A, LT Lutron, Coopersburg, PA, USA; band pass 260–390 nm), which is appropriate for UVB detection. To account for spatial variation within the cage, UV intensity was measured at 6–8 different locations at the animal level, and the average value was used for dose calculation. The UVB dose (mJ/cm^2^) was calculated by multiplying the measured UV intensity (mW/cm^2^) by the exposure time (seconds). One minimal erythema dose (1 MED) was defined as 60 mJ/cm^2^, and irradiation time was adjusted accordingly to achieve the desired dose.

Following UVB exposure, test substances were intradermally injected once into a 7 cm^2^ dorsal area at 100 µL per site, totaling seven injections per rat. The negative and UVB control groups received saline, the test groups received DHC, and the comparative control groups received PLLA and HA, respectively. All products were administered at the same injection volume and used as provided, in accordance with the manufacturers’ instructions.

All animals were euthanized by carbon dioxide (CO_2_) inhalation following the recommendations of the AVMA Guidelines for the Euthanasia of Animals (2020) [34]. CO_2_ was delivered into the chamber at a controlled fill rate of 30–70% of the chamber volume per minute to reduce potential distress. Throughout the procedure, the animals were continuously observed, and euthanasia was confirmed once both respiration and cardiac activity had ceased. This technique was selected because it is broadly accepted as an effective method that minimizes discomfort in laboratory rodents.

#### 4.3.2. Quantification of Antioxidant Enzymes, Pro-Inflammatory Markers, and Wrinkle-Related Proteins

Three weeks after treatment, tissue samples were collected and homogenized using a TissueLyser II (Qiagen) and centrifuged at 2000× *g* for 10 min. The resulting supernatant, with protein concentration determined using the BCA Protein Assay Kit, was used for subsequent analyses:

- Evaluation of antioxidant enzyme activity: Activities of SOD and CAT were measured according to the protocol described in Section 4.2.5.
- Evaluation of pro-inflammatory and wrinkle-related proteins expressions: ELISA assays were conducted following the same protocol using: Rat IL-6 Quantikine ELISA Kit (R6000B, R&D Systems), Rat TNF-α ELISA Kit (KRC3011, Invitrogen, Carlsbad, CA, USA), IL-1 Beta Rat ELISA Kit (Ab100768, Abcam), Rat Collagen Type I ELISA Kit (ab285314, Abcam).

#### 4.3.3. Histological Evaluation of Skin Tissue

Skin samples collected 3 weeks post-experiment were fixed in 10% formalin for ≥24 h, paraffin-embedded, sectioned, and subjected to HE, MT, and FM staining to evaluate epidermal thickness, collagen deposition, and melanin production, respectively.

- For HE staining, sections were stained with hematoxylin (S3309; Dako, Glostrup, Denmark) and eosin (318906; Sigma). Images of the epidermis and papillary dermis were captured at 400× magnification using a light microscope (MD3000LED; Leica, Wetzlar, Germany), and epidermal thickness was quantified using ImageJ (NIH). Increased thickness indicated UV-induced photoaging.
- For MT staining, sections were sequentially treated with Bouin’s solution, Weigert’s iron hematoxylin, Biebrich scarlet–acid fuchsin, phosphomolybdic–phosphotungstic acid, and aniline blue. Collagen fiber density (%) was quantified using Zen software (ZEN 3.4 (blue edition), Carl Zeiss Microscopy GmbH, Jena, Germany) as the ratio of collagen fiber area (blue) to the total papillary dermis area, where higher density reflected greater collagen deposition.
- For FM staining, sections were processed using the Fontana Masson Stain Kit (ab150669; Abcam) according to the manufacturer’s instructions. Melanin-positive areas (black pigmentation) were quantified with ImageJ, expressed as melanin ratio (%), with higher values indicating greater melanin accumulation.

#### 4.3.4. Skin Measurement: Were Performed at 2 and 3 Weeks After Injections

Wrinkle depth measurement: Under anesthesia, a silicone polymer (Silflo, Alsip, IL, USA; Flexico Developments) was applied to the dorsal skin of rats to obtain skin replicas after 5-min curing period. Wrinkle morphology and average wrinkle depth (μm) were quantified using a Visioline (VL650; Courage + Khazaka electronic GmbH, Köln, Germany). A smoother surface with fewer pronounced wrinkles indicates a decrease in wrinkle depth.

Skin elasticity measurement: Skin elasticity was evaluated using a Cutometer (Dual MPA580; Courage + Khazaka electronic GmbH) under a negative pressure of 300 mbar with 2 s suction/relaxation cycles repeated three times per site. Skin deformation was recorded via infrared detection using a 2 mm probe, and the R2 (gross elasticity) parameter was calculated. Values closer to 1 indicate greater skin elasticity and resilience.

TEWL Assessment: TEWL was measured using a Tewameter (TM300; Courage + Khazaka electronic GmbH). Results were expressed in g/m^2^/h, with lower values indicating enhanced skin barrier integrity.

Skin hydration measurement: Skin hydration was measured using a Corneometer (CM825; Courage + Khazaka electronic GmbH), which measures moisture via electrical capacitance. Results are reported in arbitrary units (AU), with higher values indicating better hydration.

### 4.4. Statistical Analysis

For both in vitro and in vivo studies, we performed statistical analysis using IBM SPSS Statistics 27.0 software, and graphed using GraphPad Prism 10.2.2. Data were presented as mean ± SD. Normality test was performed for each measurement, and an independent *t*-test (parametric method) or a Mann–Whitney U test (non-parametric method) was applied accordingly to determine statistical significance. The significance threshold was determined at *p* < 0.05.

## 5. Conclusions

This study demonstrates that DHC, a dual hyaluronic acid hybrid complex integrating LMW- and HMW-HA within a minimally cross-linked matrix, exhibits broad preclinical bioactivity across multiple models relevant to skin aging. In controlled in vitro systems and a UVB-induced photoaging animal model, DHC enhanced fibroblast viability and extracellular matrix production, improved antioxidant capacity and barrier function, and attenuated inflammatory responses and melanogenesis without inducing overt pro-inflammatory effects. These findings suggest that the hybrid HA architecture of DHC supports synergistic biological effects extending beyond simple volumization or hydration. Importantly, the present results should be interpreted as evidence of preclinical biological potential rather than clinical efficacy. While the observed effects support the concept of DHC as a low-inflammatory, HA-based biostimulatory platform, further studies incorporating mechanistic pathway validation, formulation-matched comparators, human ex vivo skin models, and well-designed clinical trials are required to determine durability, safety, and translational relevance in clinical practice. Collectively, this work provides a foundational preclinical framework to support future translational investigation of DHC as a novel HA-based biomaterial.

## Data Availability

The raw data supporting the conclusions of this article will be made available by the authors on request.

## Supplementary Materials

The following supporting information can be downloaded at https://www.mdpi.com/article/10.3390/ijms27021027/s1.

## Abbreviations

The following abbreviations are used in this manuscript:

AU	Arbitrary unit
BDDE	1,4-butanediol diglycidyl ether
CaHA	Calcium hydroxyapatite
CAT	Catalase
CCL1	Chemokine (C-C motif) ligand 1
DHC	Dual hyaluronic acid compound
ECM	Extracellular matrix
FM	Fontana-Masson
HA	Hyaluronic acid
HDF	Human dermal fibroblasts
HE	Hematoxylin and Eosin
HMW-HA	High-molecular-weight HA
IL	Interleukin
LMW-HA	Low-molecular-weight HA
LPS	Lipopolysaccharide
MITF	Microphthalmia-associated Transcription Factor
MT	Masson’s Trichrome
NO	Nitric oxide
PDLLA	Poly-D, L-lactic acid
PLLA	Poly-L-lactic acid
SA-β-gal	Senescence-Associated beta-Galactosidase
SOD	Superoxide dismutase
TEWL	Transepidermal water loss
TNFR-2	Tumor Necrosis Factor Receptor 2
TNF-α	Tumor Necrosis Factor-alpha
TRP1	Tyrosinase-related protein 1
TYR	Tyrosinase
UVB	Ultraviolet B
α-MSH	Alpha-melanocyte-stimulating hormone
