# Supplementary material for: Regenerative Skin Remodeling by a Dual Hyaluronic Acid Hybrid Complex in Multimodal Preclinical Models
Source: Int J Mol Sci. 2026 Jan 20;27(2):1027. doi: 10.3390/ijms27021027 (PMC12841826; doi:10.3390/ijms27021027)
Supplement: Supplementary file 1 [file ijms-27-01027-s001.zip › ijms-4063652-supplementary.pdf]

Supplementary Materials

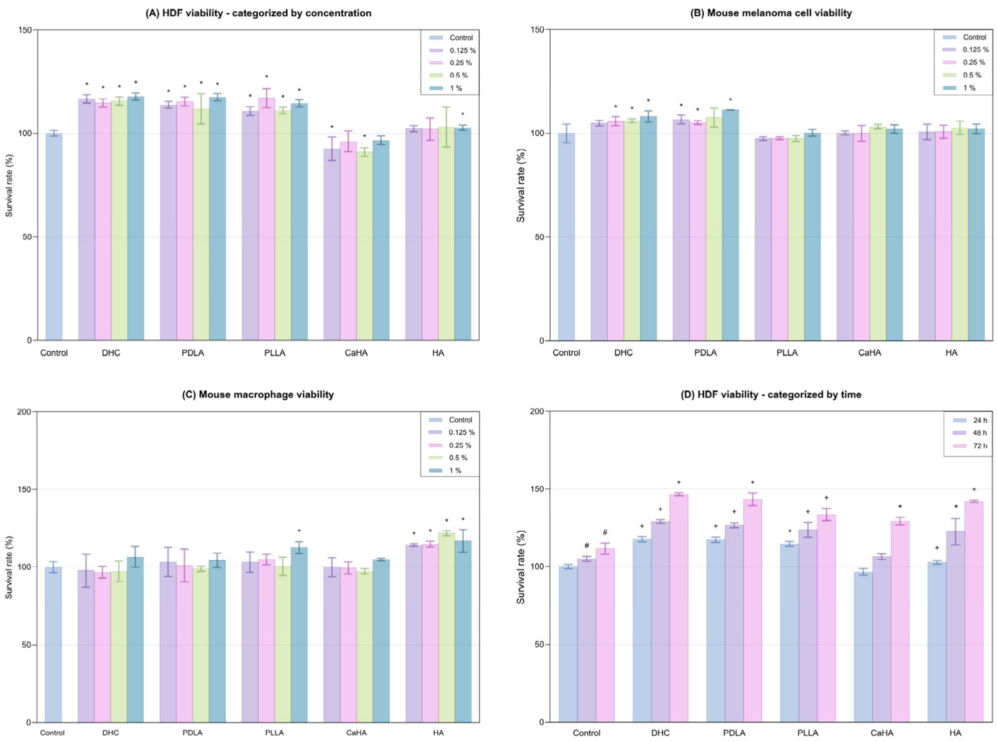

Figure S1. Cell viability of DHC and other comparators. (A) HDF viability categorized by the concentration of test products (0.125–1%). (B) Mouse melanoma cell viability. (C) Mouse macrophage viability. (D) HDF viability categorized by incubation time (24, 48, 72 h) with the test products at 1% concentration. Values are expressed as mean ± SD. *$p < 0.05$ vs control; #$p < 0.05$ vs. control at 24h; +$p < 0.05$ vs. control at the same time point.
